# Swift metabolite changes and leaf shedding are milestones in the acclimation process of grapevine under prolonged water stress

**DOI:** 10.1186/s12870-019-1652-y

**Published:** 2019-02-11

**Authors:** Asfaw Degu, Uri Hochberg, Darren C. J. Wong, Giorgio Alberti, Naftali Lazarovitch, Enrico Peterlunger, Simone D. Castellarin, Jose C. Herrera, Aaron Fait

**Affiliations:** 1 The French Associates Institute for Agriculture and Biotechnology of Drylands, The Jacob Blaustein Institute for Desert Research, Ben-Gurion University of the Negev, Sede Boqer campus, Midreshet Ben Gurion, Israel; 2 College of Agriculture and Environmental Sciences, Bahir Dar University, Bahir Dar, Ethiopia; 3 Department of Agricultural, Food, Environmental and Animal Sciences, University of Udine, Udine, Italy; 4 Intitute of Soil, Water and Environmental Sciences, Agricultural Research Organization Rishon LeZion, Rishon LeZion, Israel; 5 Wine Research Centre, The University of British Columbia, Vancouver, Canada; 6 Division of Viticulture and Pomology, Department of Crop Sciences, University of Natural Resources and Life Sciences Vienna (BOKU), Tulln, Austria

**Keywords:** Grapevine, Metabolite alteration, Transcript alteration, Leaf shedding, Water stress, Grapevine acclimation

## Abstract

**Background:**

Grape leaves provide the biochemical substrates for berry development. Thus, understanding the regulation of grapevine leaf metabolism can aid in discerning processes fundamental to fruit development and berry quality. Here, the temporal alterations in leaf metabolism in Merlot grapevine grown under sufficient irrigation and water deficit were monitored from veraison until harvest.

**Results:**

The vines mediated water stress gradually and involving multiple strategies: osmotic adjustment, transcript-metabolite alteration and leaf shedding. Initially stomatal conductance and leaf water potential showed a steep decrease together with the induction of stress related metabolism, e.g. up-regulation of proline and GABA metabolism and stress related sugars, and the down-regulation of developmental processes. Later, progressive soil drying was associated with an incremental contribution of Ca^2+^ and sucrose to the osmotic adjustment concomitant with the initiation of leaf shedding. Last, towards harvest under progressive stress conditions following leaf shedding, incremental changes in leaf water potential were measured, while the magnitude of perturbation in leaf metabolism lessened.

**Conclusions:**

The data present evidence that over time grapevine acclimation to water stress diversifies in temporal responses encompassing the alteration of central metabolism and gene expression, osmotic adjustments and reduction in leaf area. Together these processes mitigate leaf water stress and aid in maintaining the berry-ripening program.

**Electronic supplementary material:**

The online version of this article (10.1186/s12870-019-1652-y) contains supplementary material, which is available to authorized users.

## Background

Alteration in global climate is projected to intensify the incidence of drought worldwide [1, 2]. Climate model-based predictions suggest an increase in average temperature around the globe [3, 4]. Seasonal drought and high summer temperatures have been increasingly affecting the viticulture industry worldwide, given the negative association between water scarcity and grapevine growth, productivity and quality [5–10]. Such trend implies severe consequences when considering that two-thirds of world’s viticulture regions have annual rainfall of less than 700 mm, i.e. below full crop evapotranspiration [11]. Grapevine (*Vitis vinifera* L.) is considered suited to Mediterranean like climates [12]. Grapevine adjust its cellular homeostasis under stress conditions via a reprogramming of metabolism and cellular physiology [8, 10, 13–22], osmotic adjustment [23, 24] and improved ROS tolerance [25, 26] Previous study on stress responses at the whole-plant level has shown that water stress-induced leaf shedding was preceded by petiole cavitation before stem cavitation occurred [27]. With this respect grapevine petiole is more vulnerable to drought-induced embolism than the stem [28], leading to leaf shedding at sever stress conditions.

In spite of the numerous recent studies on grapevine response to stress (reviewed by [29, 30]), few have explored the leaf long-term molecular acclimation to stress in the field [31, 32]. It is assumed that physiological acclimation alleviates the effect of water deficit and allows plant productivity [23, 33]. Our previous report on Merlot grapevine showed that deficit irrigation resulted in altered berry metabolism [34]. Here combining plant physiology with metabolomics and RNAseq analysis we characterized the response of leaves to a seasonal-long drought in Merlot vines.

## Results

**Fig. 1:**
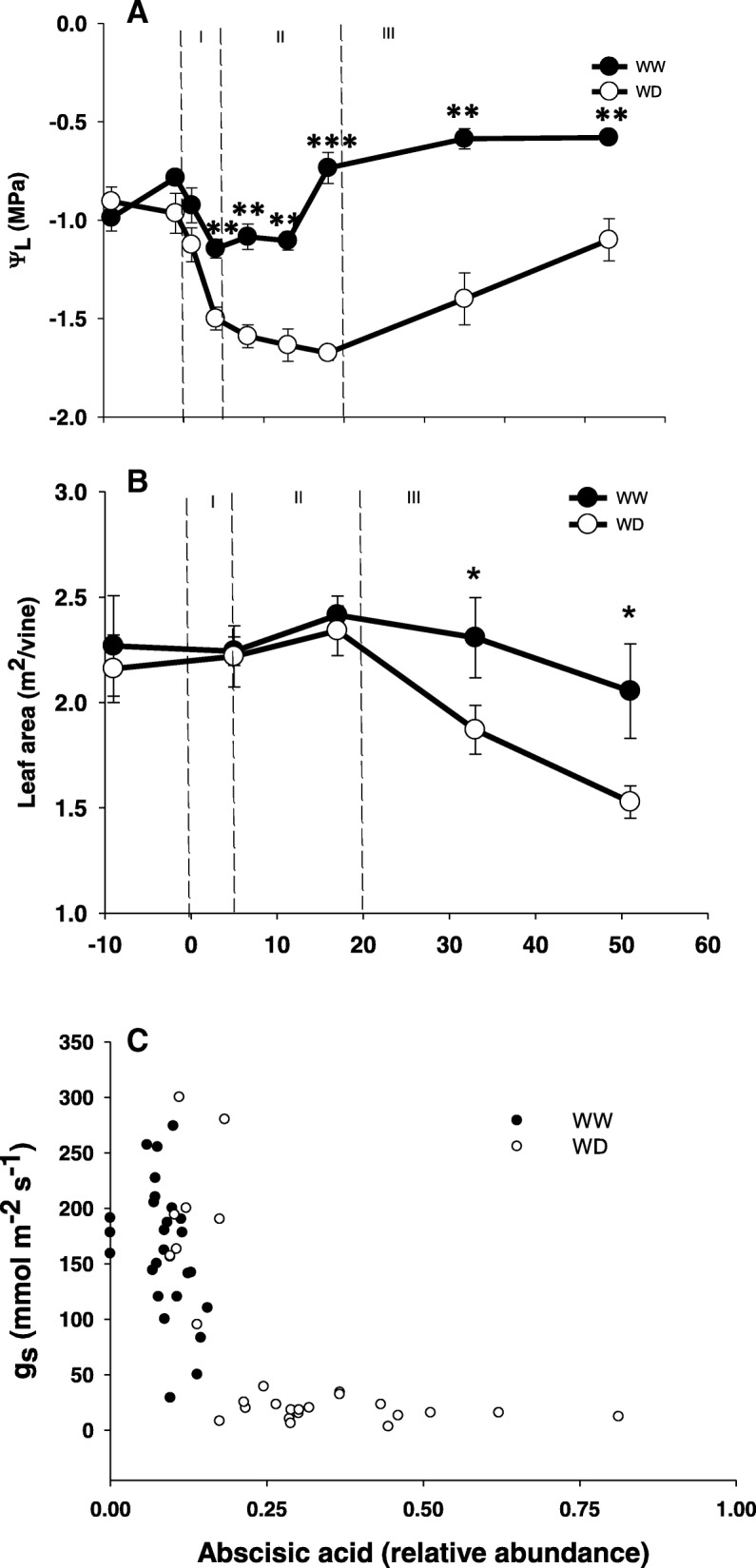
Effect of water stress on leaf water potential (Ψ_L_, MPa) (**a**), leaf area of vines (**b**) and the relationship between stomatal conductance and ABA relative abundance (**c**) during the course of the experiment. Bars at each time point represent S.E. (*n* = 4). Asterisks indicate statistically significant differences between treatments according to student t-test (*P* < 0.05)

**Fig. 2:**
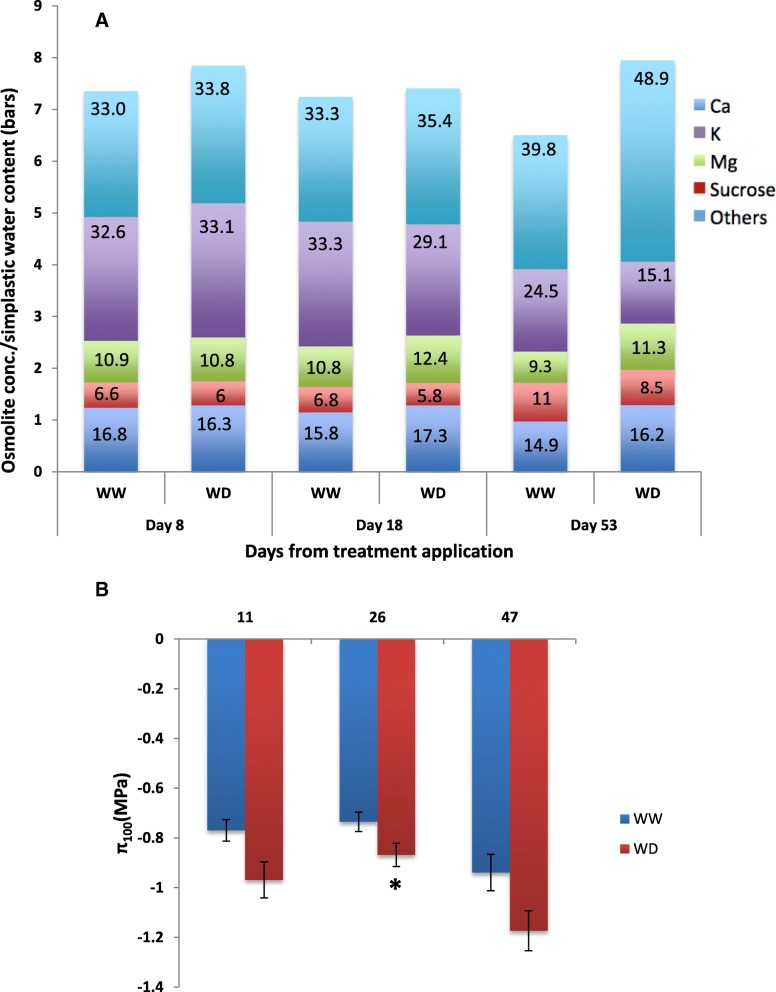
**a**) Solutes contribution (calculated as percentage) to the osmotic potential of leaves under well-watered (WW) and water deficit (WD) conditions after 8, 18 and 53 days of irrigation treatments imposition. **b**) The osmotic potential at full turgor (π_100_) of WW and WD treated vines as derived from pressure-volume curve analysis after 11, 26 and 47 days after irrigation treatments imposition. Asterisks indicate significant differences between treatments according to student’s t-test (*P < 0.05, **P ≤ 0.01, ***P < 0.001)

Leaf metabolic and physiological responses of Merlot grapevine showed different temporal pattern of changes during the long-term deficit irrigation: (1) In the very first days (i.e. 0 to 5 DAT) a swift alteration in the leaf metabolism and transcriptional program occurred in parallel to a steep decline in stomatal conductance and leaf water potential (Fig. 1a), (2) a transient settling of the leaf physiology was recorded, i.e. leaf water potential reached a low steady-state level (i.e. 5–20 DAT); (3) a gradual reduction in total leaf area (Fig. 1b) and increased accumulation of osmolites leading to a leaf osmotic adjustment were accompanied by the reverse pattern of change in leaf water potential and the lessening of metabolic perturbation. This period is referred to the stress relaxation period (SRP, from 20 DAT onwards). The three major temporal patterns of water stress response are described below in details.

### Leaf hydraulic and osmotic adjustments

Expectedly, a marked reduction in stomatal conductance (g_s_) was observed already within four days from deficit irrigation imposition (Additional file 1: Table S1). In association with the decrease in soil water content [23], stomatal conductance of water stressed vines eventually reached the lowest point at day 8 and remained at that level thereafter (15–20 mmol H_2_O m^− 2^ s^− 1^) (Additional file 1: Table S1A). The decrease in g_s_ at "severe" water stress condition was highly associated with a corresponding increase in leaf abscisic acid (ABA) amount (Fig. 1c). The leaf water potential (Ψ_l_) exhibited three distinct temporal pattern of changes across the stress period: a progressive decline from − 0.9 MPa to − 1.6 MPa within the very first days of the experiment (Fig. 1a), followed by a period of stable low values (− 1.6 up to − 1.67 MPa) until day 20, and finally a gradual increase up to − 1.1 MPa during the third phase of response towards harvest.

Leaves from water deficit vines resulted in more negative osmotic potential at full turgor (− 1.17 MPa) than the control plants (− 0.94 MPa) (Fig. 2b) likely due to higher concentration of solutes (Fig. 2a). As the stress progressed, the role of K^+^ in osmoregulation declined its contribution from 33.1% at day 8 to 15% at day 53 of the treatment time while the contribution of Ca^2+^ and sucrose increased from 33.8% to 48.9 and 5.9 to 8.9%, respectively, at the aforementioned days (Fig. 2a). At the end of the experiment, the mineral ions Ca^2+^, K^+^ and Mg^2+^ contributed 48.9, 15 and 11.3%, respectively, to the osmoregulation in WD leaves followed by sugars (8.6%) (Fig. 2a).

### Decreased leaf transpiration and metabolic reorganization: The very first lines of response to deficit irrigation

Large metabolic perturbations characterized the first 20 days from the start of the deficit irrigation. A prominent significant perturbation in the leaf metabolism at 4 DAT (Additional file 2: Figure S1), with the change in content of 40% of the measured metabolites, is followed by an intermediate phase where the changes settle at about 25% in association to a lessened alteration in leaf physiology (as mentioned above).

**Fig. 3:**
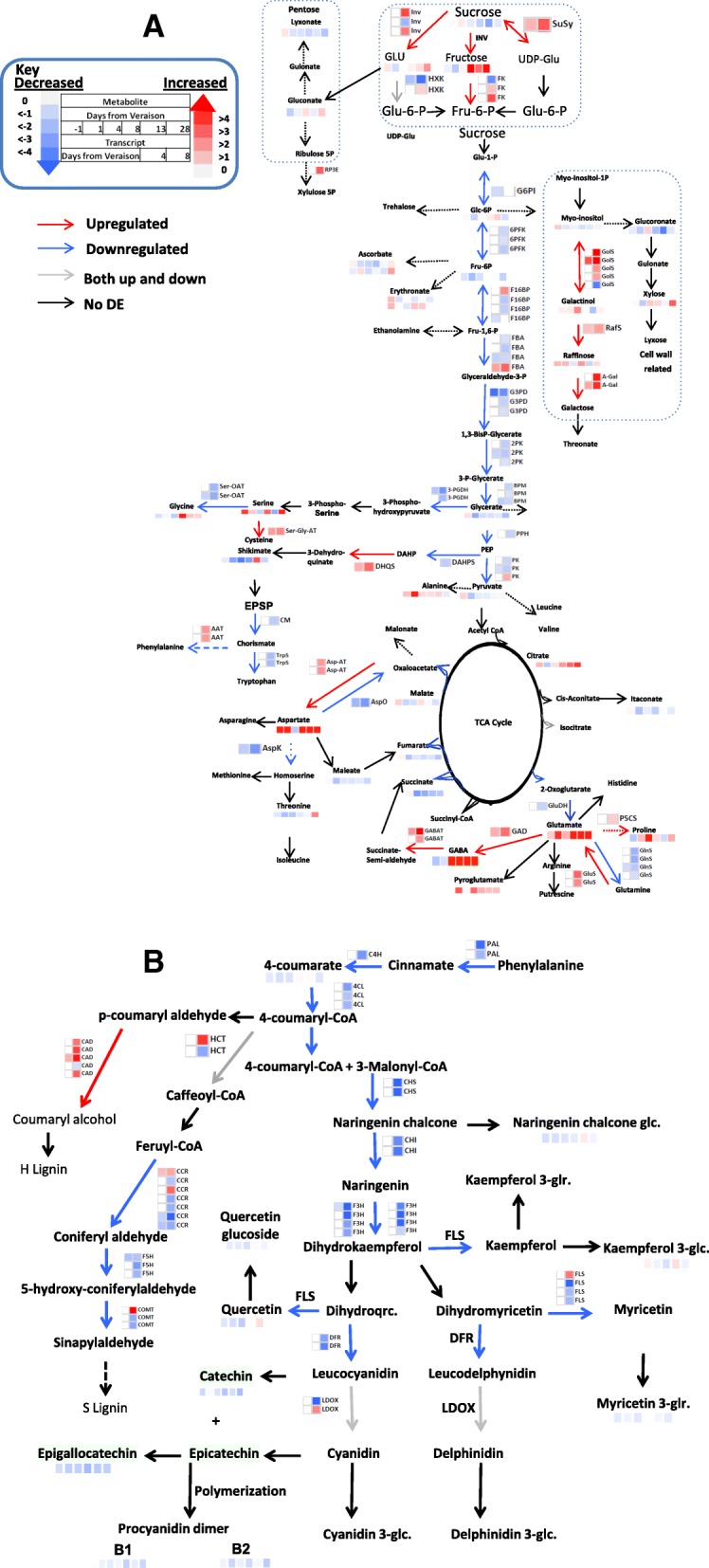
Heat map of central (**a**) and secondary (**b**) metabolites and respective gene transcripts changes under water stress. Each box of the heat map in transcript changes due to WD (heat map at the side of each arrow) are presented as log2 fold-change ratio from the average of control plants. Three biological replicates were used to generate mean value at each transcript time point. Each box of the heat map displayed at the end of each metabolite pathway arrow represents the metabolite fold-change (Treatment/Control). The metabolite fold-changes were calculated on mean values of four biological replications. Increase (red) and decrease (blue) are presented in the color scale for both transcript and metabolite changes

In water stressed vines, amino acids, which showed major changes in response to stress, followed different patterns of change. First, a reduction in glycine (1.23-fold) and gamma aminobutyric acid (GABA) (1.56-fold) during the early phase of stress (first 4 days) was measured. During the intermediate period a transient increase in threonine (2.2-fold), glutamate (2-fold), proline (+CO2) (3.5-fold) and pyroglutamate (1.8-fold) was recorded. Prolonged water stress conditions (after day 20) caused strong progressive increase in GABA (up to 17-fold), leucine (2-fold) and aspartate (up to 4.5-fold) (Fig. 3a).

Almost all of the annotated sugars, except for galactinol and raffinose, showed a marked decrease during the first phase of response to the stress particularly at 4 DAT, probably as a result of reduced net photosynthesis [34]. In contrast, the stress related oligosaccharides, galactinol (up to 1.5-fold) and raffinose (up to 1.5-fold) accumulated throughout the experiment. The intermediate phase was characterized by a progressive decline in the level of sucrose (2-fold), but a reversed pattern of change of sucrose derived sugars, e.g., glucose (up to 1.4-fold) and fructose (up to 2-fold) which increased in relative content from 8 DAT onwards (Fig. 3a). A progressive increase in the level of citrate (up to 2-fold) in water stressed vines was also measured during the early, mid and late phase of the stress. On the contrary, most of the other organic acids exhibited a general mild reduction during the mid (day 8 and 13) and late phase of the stress (Fig. 3a).

Among the flavonoid compounds only few were altered by the stress. Flavanols such as epicatechin and epigallocatechin, and the shikimate pathway intermediate, shikimate, were decreased consistently over the experiment period (Fig. 3 a and b; Additional file 1: Table S2), but overall reduction level remained mild. Similar to the pattern observed in organic acid, most hydroxycinamate compounds (cinnamate (4-hydroxy), cinnamate (3-hydroxy), caffeate (cis), caffeate (trans), quinate (3-caffeoyl cis)), kampferol (+H2) and phosphoric acid showed a marked reduction during the early stress period followed by a slight increase at later stage of the stress (Additional file 1: Table S3).

The stress relaxation period (20 DAT onwards) was characterized by a reduction in the vine total leaf area (by 28.6% than the control vines) (Fig. 1b) in the deficit irrigated plants. Consequently, the vine improved its water balance by reducing the evaporative surface, allowing the increase of the water potential and relive the signs of metabolic stress [34, 35].

### Alteration of leaf transcripts in response to water deficit

**Fig. 4:**
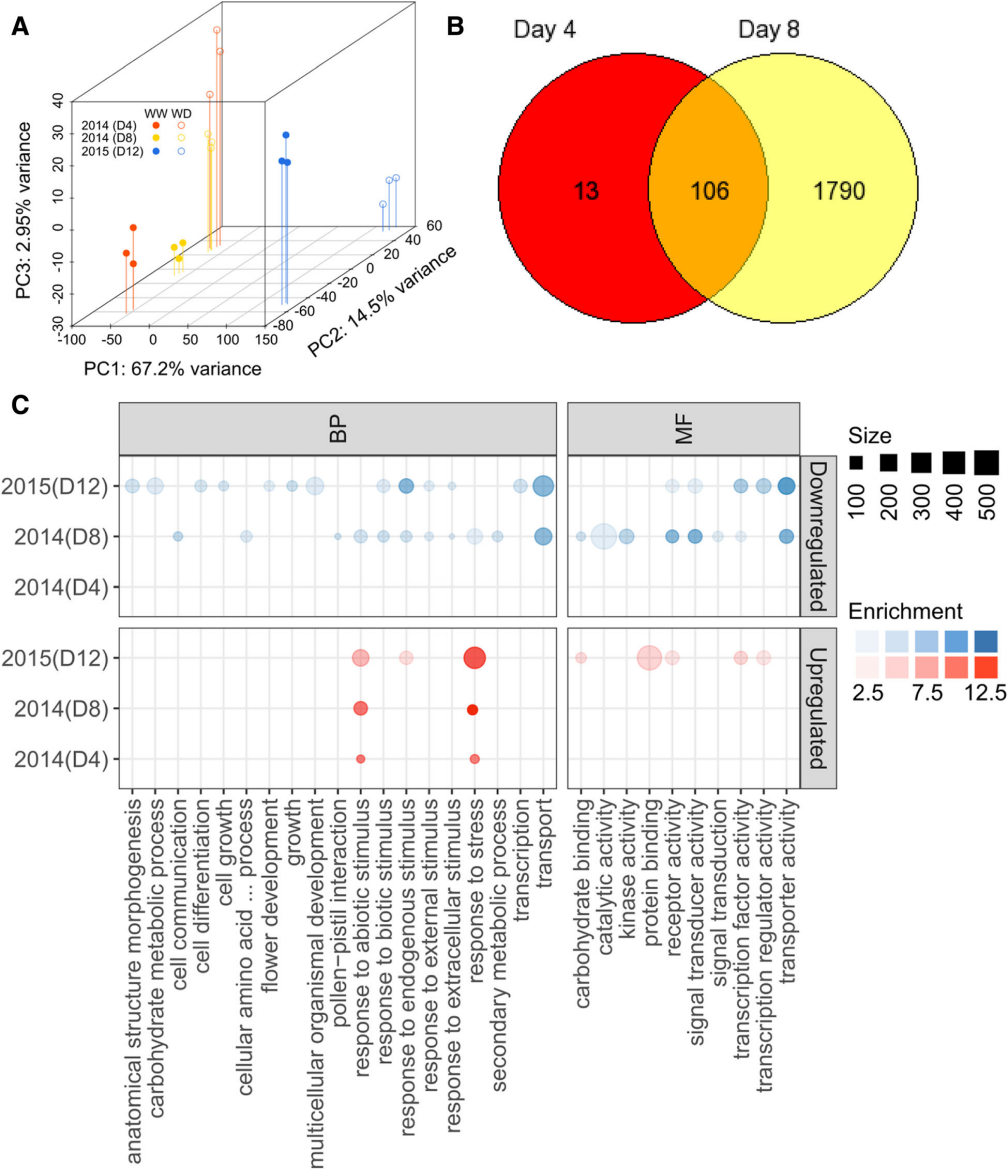
Leaf transcript profiles of Merlot vines under well-watered (WW) and water deficit (WD) conditions. **a**) PCA of transcript changes at day 4 (D4), 8 (D8) (2014) and 12 (D12) (2015) after deficit irrigation imposition. Filled circles indicate WW and open circles WD treatment. **b**) The Venn diagram representing common and unique genes from the total DE (FDE < 5%, |log2Fc| ≥1) genes at day 4, 8 (2014) and day 12 (2015). Gold, red and blue colors are day 4, 8 (2014) and day 12 (2015), respectively. **c**) Summary of enriched plant gene ontology SLIM biological process (BP) and molecular function (MF) terms associated with the DE genes (FDE < 5%, |log2Fc| ≥1) at day 4, 8 (2014) and day 12 (2015). The size and opacity of each circle indicate the number of genes and enrichment score (−log10(FDR)) of each enriched category, respectively. Large circle indicate more genes assigned to each category. Darker opacity of blue (downregulated) and red (upregulated) colors indicate stronger enrichment values and vice versa for lighter colors

Using RNAseq analysis, an average of 14.8 and 29.0 million single-end reads were successfully aligned and summarized in sequenced samples of 2014 (4 and 8 DAT) and 2015 (12 DAT), respectively (Additional file 1: Table S4). The PCA plot generated using the entire expressed transcript dataset (Additional file 1: Table S5) showed clear separation of the water deficit treatments (explaining ca. 15% of the variation) in the second principal component (PC2) despite having the majority of variation (ca. 67%) contributed by difference in vintages (2014 vs 2015) in PC1 (Fig. 4a). This is not unexpected given that recent transcriptome insights from grapevine genotype x environment experiments showed that developmental stage and vintage conditions are two key variable that influences transcriptional variation at least in the berry [36]. As such, the large variation observed in PC1 may be due to the differences in the climactic parameters of the two vintages.

Notwithstanding this, a greater dispersion of the data points was still observed when the vines were exposed to longer water deficit (Fig. 4a), which reflected the extent of differentially expressed genes (FDR < 0.05; > 2-fold change) at each sampling time, i.e. 119, 1896 genes at day 4 and 8 of 2014, respectively (Additional file 1: Table S6 – S8). When comparing within each list of DEGs, 106 DE gene transcripts were common for both sampling times (Fig. 3b, Additional file: Table S9). Thirteen, and 1790 were specific to day 4 and 8 DAT, respectively. Among the thirteen genes specific to the early stress response (4 DAT), VIT_11s0052g01650 (signaling pathway), VIT_16s0022g00890 (cell wall metabolism), VIT_06s0004g05020 (glyoxylate & dicarboxylate metabolism), VIT_07s0031g00920 (inositol, phosphate metabolism) VIT_04s0008g07110 (aspartate metabolism), and VIT_15s0046g01560 showed up-regulation by WD while the other seven genes VIT_12s0028g02200 (riboflavin metabolism), VIT_06s0061g00100 (starch and sucrose metabolism), VIT_05s0049g00930 (inorganic phosphate transporter), VIT_08s0007g01940 (glycerolipid metabolism), VIT_17s0053g00070 (metal-nicotianamine transporter), VIT_12s0059g01400 (tyrosine specific protein phosphatase), and VIT_03s0017g02110 (anthocyanin biosynthesis) were down-regulated by water deficit (WD). Figure 4c shows the gene ontology enrichment analysis with the different representative categories. The early stress period (day 4) mainly leads to activation of stimuli perception associated responses. As the stress progressed a wide range of stress related transcript including response to abiotic stimulus, response to stress, response to endogenous stimulus, cellular process related to cell death and response to biotic stimulus were strongly induced.

Among the metabolic genes, transcripts involved in sucrose metabolism showed a trend of up-regulation in the early response to stress (Fig. 3a, Additional file 1: Tables S6 – S8). Genes transcripts coding for the conversion of sucrose to glucose, invertase (VIT_05s0077g00510 2-fold, VIT_13s0074g00720), fructose (invertase) and UDP-glucose (VIT_07s0005g00750 1.7-fold) were up regulated particularly at 8 DAT of the first season consistent with changes at 12 DAT in the second season in concomitance with a decrease of downstream genes of the glycolysis. The transcript data matched the observed declining level of sucrose towards harvest and the accumulation of secondary sugars. Notably, only seven transcripts from the glycolysis pathway were affected by water stress at day 4 of the experiment.

In the PPP (Pentose Phosphate Pathway), three transcripts (VIT_01s0146g00060, VIT_14s0030g01900, VIT_15s0048g00370) involved at different steps of the pathway were found to be significantly down-regulated at later stage of the stress. Ribulose phosphate 3-Epimerase (VIT_04s0043g00310), which is known for its role in mitigating oxidative stress [37, 38], on the other hand, was up-regulated (2.5-fold) under prolonged water limitation (day 8) (Fig. 3a, Additional file 1: Tables S6 – S8). The TCA cycle gene transcripts were not markedly altered in the first four to eight days of the water deficit in line with the overall mild changes in the TCA intermediates (Fig. 3a, Additional file 1: Tables S6 – S8).

Changes in amino acid metabolism were primarily shown at 8 DAT with the exception of dehydroquinate synthase (DHQS) (Fig. 3a). In the shikimate pathways, down-regulation in chorismate synthase (CM) and tryptophan synthase (TrpS) at 8 DAT (1.6 fold-change), was coupled with the upregulation of prephenate dehydratase (ADT/PDT) (4.4-fold) which makes Phe from prephenate in two steps (except for one ADT/PDT - VIT_06s0061g01300).

The aspartate aminotransferase (AAT) transcripts were commonly up-regulated at 8 DAT in 2014 (Fig. 3a, Additional file 1: Table S3). This was coupled with significant down-regulation of Asp oxidase (AspO), which breaks down Asp back to Oxaloacetate (OAA), and Asp kinase (AspK), which initiates the biosynthesis of Met, Lys, and Thr amino acids, suggesting that increased levels of Asp were due to a reduction of its breakdown.

Glutathion related transcripts, glutamine synthase (GlnS) (VIT_01s0011g02200, VIT_07s0104g00610, VIT_14s0006g00350, VIT_17s0000g01910), were down-regulated (2.1-fold) at 8 DAT, while glutamate synthase (GluS) (VIT_16s0098g00290, VIT_15s0024g01030) increased (2.4-fold) (Fig. 3a, Additional file 1: Tables S6 – S8), in support of the accumulation of Glu in 2014. Notably, Glutamic acid decarboxylase (GAD, VIT_17s0000g00920), was up-regulated (2.2-fold) at 8 DAT, consistent with the accumulation of GABA level as early as 4 DAT and the induction of GABA-T.

Glu derived stress amino acid, proline, which accumulated together with GABA already at 4 DAT, is synthesized by the enzyme delta 1-pyrroline-5-carboxylate synthase (VIT_13s0019g02360) which was up-regulated (1.3-fold) concomitantly with a down-regulated Pro degradation at 8 DAT (Fig. 3a, Additional file 1: Tables S6 – S8).

### Up-regulation of ABA biosynthesis

A large number of transcripts encoding for ABA metabolism were up-regulated under water stress at both 4 and 8 DAT. For example, two out of three transcripts encoding the key regulatory enzyme, 9-cis-epoxycaoteniod dioxygenase (NCED, VIT_02s0087g00930 and VIT_19s0093g00550) [39] and one ABA β-glucosidase (VIT_17s0000g02680, hydrolyzes conjugated ABA-glucose to active abscisate) (up to 6-fold) where significantly up-regulated at 4 and 8 DAT (Additional file: Tables S6, S7, S8), supporting the concurrent higher ABA level in water-stressed vine leaves (Additional file 1: Table S2). On the other hand, transcripts encoding for CYP707A, e.g. VIT_18s0001g10500 and VIT_03s0063g00380 (involved in ABA catabolism) have shown down-regulation (2.3-fold) under stress indicating that the higher accumulation of ABA under water stress is likely due to its induced biosynthesis and reduced catabolism. Concomitantly, the transcript encoding for SNF1 protein kinase 2–3/AKIP/OST1 (VIT_07s0197g00080), which is involved in controlling stomata aperture under drought stress [40, 41] showed an up-regulation (5.7-fold) in water deficit vines.

### ROS metabolism

In the current study we observed large number of oxidative stress related genes differentially expressed during longer water stress in 2014 (Additional file 1: Tables S6 – S8). A gene transcript coding for ascorbate peroxidase (VIT_08s0040g03150), enzyme involved in H_2_O_2_ removal, up-regulated at 8 DAT in 2014, while the other transcript (VIT_06s0004g03550) showed a down-regulation. Moreover, a gene transcript coding for ascorbate dehydroredactase (VIT_00s0317g00050 and VIT_00s0317g00040), which catalyze dihydroascorbate back to ascorbate was up-regulated. In line with this results, as the water stress progressed over time (8 DAT), accumulation of related ascorbate, and dehydroascorbate were evident (Additional file 1: Tables S6 – S8). We found gene transcripts (VIT_04s0008g06780 and VIT_02s0025g03590 - glutathione peroxidase, VIT_07s0104g01400, VIT_07s0104g01420, VIT_14s0068g01570, VIT_14s0068g01570, VIT_18s0001g04600, VIT_10s0003g00390 - glutaredoxin, VIT_07s0130g00220, VIT_07s0130g00220, VIT_07s0130g00220 - Peroxidase_class_III) commonly involved in the glutathione metabolisms up-regulated in 2014 at the later stage of the stress (8 DAT) (Additional file 1: Tables S6 – S8).

### Changes in flavonoid and lignin biosynthesis under water stress

Gene-transcripts and associated metabolites of the flavonoid pathway showed a general mild reduction under WD (Fig. 3b). Among the genes encoding for phenylalanine ammonia lyases (PAL), VIT_06s0004g02620 was down-regulated (> 3-fold) during late stress period. In agreement with the metabolite response in the phenylpropanoid pathway, transcripts encoding for anthocyanidin reductase (ANR) (VIT_00s0361g00040) were highly down-regulated at 8 DAT (> 4-fold). Consistently, VviMYBPA1 (VIT_15s0046g00170), the transcriptional regulator for both ANR and leucoanthocyanidin reductase (LAR) [15], was significantly down-regulated at 8 DAT (Additional file 1: Tables S6 - S8). Among the lignin biosynthesis, transcripts encoding hydroxycinnamoyl-CoA shikimate/quinate hydroxycinnamoyltransferase (HCTs), cinnamoyl-CoA reductase (CCR) and caffeic acid methyltransferase (COMT) were modulated displaying mixed patterns of change. In contrast to the general trend, as the stress progressed, a pronounced increase in cinnamyl alcohol dehydrogenase (CAD) (the penultimate step of lignin biosynthesis) was observed (Fig. 3b; Additional file 1: Tables S6 – S8).

### Enriched cis-regulatory elements prioritizes transcriptional regulators of drought response

**Fig. 5:**
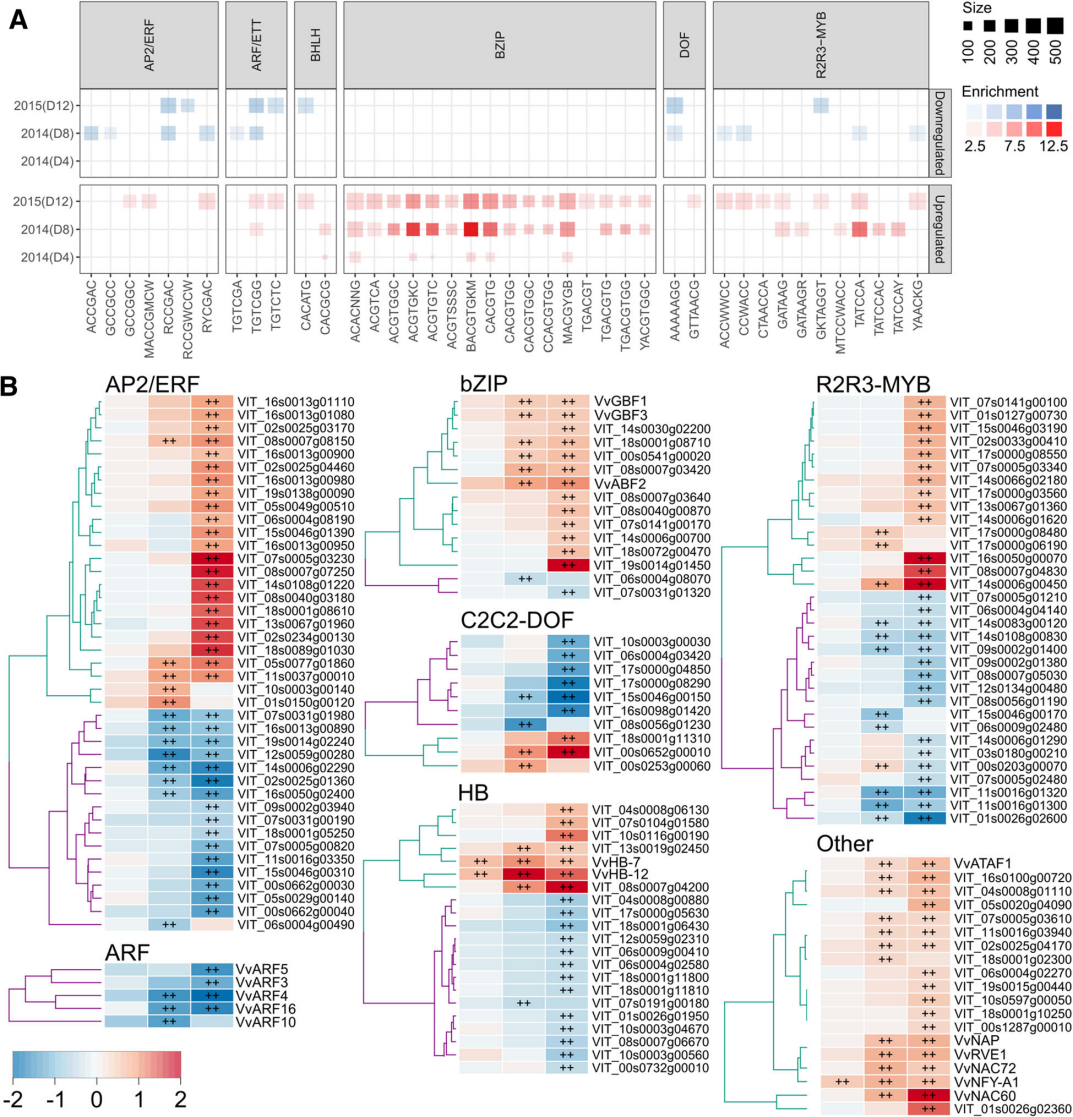
**a**) Overview of cis-regulatory elements and transcription factors involved in grapevine leaf response to water deficit. **b**) Subset of enriched cis-regulatory elements (CREs) detected in drought-modulated genes at day 4, 8 (2014) and day 12 (2015) arranged by the most common transcription factor family that recognize the enriched CRE. The size and opacity of each square indicate the number of genes and enrichment score (−log10(FDR)) of each enriched CRE, respectively. Darker opacity of blue (downregulated) and red (upregulated) colors indicate stronger enrichment values and vice versa for lighter colors

In plants, the ABA-dependent and ABA-independent signal transduction pathways largely mediates the transcriptional response to water deficit [42]. In this study, close to 20% (437 genes) of all predicted grapevine TFs [43] representing a diverse range of TF families were significantly modulated (Additional file 1: Tables S3 – S5). We observed consistent enrichment for all major ABA responsive (ABRE) and ACGT-containing elements, such as ACGTGKC, BACGTGKM, MACGYGB, and CACGTG, in the promoters of water deficit induced genes (Fig. 5a, Additional file 1: Table S8). Enrichment of these motifs were already observed at the end of early stress period (4 DAT) and as stress gets severe, more ABRE and related elements were enriched in water deficit induced gene promoters. Among the many drought-responsive bZIPs (e.g. GBF1, VIT_04s0023g01360; GBF3, VIT_15s0046g01440), the grapevine homolog of ABF2/AREB1 (VvABF2, VIT_18s0001g10450) was highly expressed and consistently upregulated (> 4-fold) when the drought was severe (Fig. 5a, Additional file 1: Table S11). The dehydration-responsive element (DRE), another critical element for regulating drought-responsive gene expression [44], was highly enriched in many drought downregulated genes, but to a lesser extent in upregulated genes. However, the major regulator for the ABA-independent pathway, DREB1A/2, were not DE in this study albeit modulation of many drought-responsive AP2/ERFs were observed. Enrichment for MYB-, HB-, and ARF-related binding sites also showed similar trends with AP2/ERF-related CREs (Fig. 5a, Additional file 1: Table S11). However, transcript expression for these TFs especially ARFs (e.g. ARF4, VIT_06s0004g03130; ARF16, VIT_06s0004g02750) were consistently downregulated in both seasons during prolonged stress while HBs (e.g. HB-7, VIT_15s0048g02870; HB-12, VIT_02s0025g02590) were upregulated in all stress periods (Fig. 5b, Additional file 1: Table S11).

## Discussion

Plant uses several coordinated responses to cope-up with drought stress [45]. The decline of leaf water potential and the subsequent decline of stomatal conductance and net photosynthesis [34] are well known stress responses [9, 46] and are thus used as a sensitive indicators for grapevine water stress. The current results (Fig. 1a, and Table S1A) and our previous report [34] link molecular responses to these physiological indicators. In Accordance with the stomatal based stress definition (moderate 50 < g_s_ < 150 or severe g_s_ < 50 mmol H_2_O m^− 2^ s^− 1^; [47]) it appears that molecular responses takes place mostly under severe stress. This was notable in ABA levels which rose only when g_s_ < 50 mmol H_2_O m^− 2^ s^− 1^ (Fig. 1c) and also in primary metabolism modification that was more notable when g_s_ were minimal (Additional file 2: Figure S1). These results confirm the notion by [48] that metabolic impairment is likely to take place only under severe stress, and that stomatal closure could buffer moderate water limitation without significant effect on the vines.

The fact that most molecular responses took place only when g_s_ were very low, undermines the importance of molecular signals in stomatal closure. Specifically, it calls into question the role of ABA that exhibited stable values during g_s_ reduction from 300 to 100 mmol H_2_O m^− 2^ s^− 1^. The same phenomenon was already observed by both [49] and [50] and supports the hypothesis that hydraulic signals close the stomata while ABA is important for maintenance of low g_s_ following water potential recovery. The same hypothesis is also supported by the recent findings of [51] that showed that most of stomatal down regulation can be explained by turgor dynamics during dehydration.

One of the interesting result in this research was that leaf shedding mediated the recovery of the vine at the physiological and molecular levels. For instance, following the marked leaf shedding event, the vines regained leaf water potential and ease the extent of metabolite perturbation. As a likely stress relaxer, this mechanism will directly reduce the evaporative surface of the vines and can also aid in redirecting photoassimilates translocation from older leaves to younger ones [52]. Coinciding with the low leaf water potential and the progressive leaf shedding, the osmotic potential (Fig. 2b) and solute concentration (Fig. 2a) showed a more negative value at later stage of the stress. It is known that the specific kind of solute involved in the osmo-regulation vary with different plant species [53–55]. Interestingly, we found that calcium was one of the most important osmolites (~ 1/3 of the total osmotic content). Differently, [24] reported that calcium ions account for less than 10% of the total osmotic content although they found a sevenfold increase in the stressed plants. Previous study indicated that idioblastic cells carrying raphides of calcium oxalate accumulated in *Vitis* leaves [56] and particularly during stress [57] probably playing a role of storage/release of calcium and thus contributing to the osmoregulation capacity of the leaves. In the current study, the higher calcium content could largely explain the difference in osmotic potential between the control and water stressed vine leaves*.* Calcium ion plays multiple roles in plant stress response such as stomatal closure [58], osmotic adjustment and leaf turgor and cell wall rigidity [24, 59], signaling molecule [60–63], improves biotic and abiotic stress tolerance of plants [64] and detoxification of heavy metals [65]. The overall elevated osmolite concentration particularly of Ca^+^ might reflect its involvement in maintaining the aforementioned cellular processes in water-stressed leaves.

Changes in metabolite level during the stress depended upon the length of imposed drought. Both the transcriptional and metabolomic data suggest the increase in stress related amino acids of the Glu family, GABA and proline (Fig. 3a). The first is part of the GABA shunt, a metabolic pathway linking Glu to the TCA cycle [66] via the entry enzyme glutamate decarboxylase, whose coding gene was upregulated early upon stress. The second is involved in the antioxidant machinery [67] and act as osmolite [68]. Its accumulation is common during incremental water deficit in plants in general [51] and in other grapevine varieties [69]. The final enzyme in proline biosynthesis (P5CS: delta 1-pyrroline-5-carboxylate synthase) mediates the rate-limiting step in the conversion of 1-Pyr-5-carboxylate to Pro. A significant up-regulation was found in P5CS while the enzyme involved in the degradation of Pro was down-regulated at 8 DAT (Fig. 2a, Additional file 1: Table S3), which supports the overall higher accumulation of Pro in water stressed vines and aligns with previous findings [8]. Moreover a greater but gradual change in N-metabolism was likely occurring during the drought period as suggested by the alteration of glutamate and aspartate and their related transcripts. Indeed, amino acid metabolism in leaves largely depends on nitrogen assimilation and de-novo amino acid metabolism, its release from protein degradation [70] and efflux into newly growing tissue [71].

Metabolic adjustments in response to stress conditions often occurs in a very short time, for example GABA metabolism can react within seconds from the beginning of the stress [72]. However, during prolonged stress periods the progressive decrease in sucrose can be attributed to a whole-plant decrease in photosynthesis rate [34] and as a consequence of earlier downregulation of cellular functions such as transport, carbohydrate metabolic process and energy metabolism. This reduction in certain cellular processes likely enable the plant to sustain key biological processes at lower rate, enhance protective metabolic responses, and limit stress related damages in growing tissues and the detrimental accumulation of ROS. Sucrose for example is degraded to downstream metabolic processes. Consequently, sucrose derived sugars (e.g., glucose, fructose, xylose and raffinose) showed reverse pattern of change. These low-molecular weight carbohydrates (glucose, fructose, galactinol and raffinose) are known compatible solutes [73] and likely involved in cellular osmotic adjustment [74] since their biosynthesis is largely induced in stressed vines compared to the control, but can also serve as protectants of cellular structures against desiccation events (e.g. during seed desiccation, [66]) and as transient carbon-storage [75]. A general downregulation in energy producing TCA cycle activity, consistent with a mild reduction in the TCA cycle intermediates was measured in response to stress (Fig. 3a). Outstandingly, a progressive increase in the level of citrate in water stressed vines was measured in the current experiment and in previous works [69]. Once synthesized in the mitochondria, citrate is either transported to the cytosol and stored in the vacuole or converted to iso-citrate via aconitase [76, 77]. However, under stress, reactive oxygen species (ROS) build up [78] and can inhibit aconitase driven citrate conversion [79] leading to the observed accumulation. Consistent to this hypothesis, the transcriptional induction of oxidative stress related genes may partly explain the accumulation of citrate under water stress (Additional file 1: Table S3).

The role of ABA in plant responses associated with stress signaling and transduction pathways is well described in the plant literature [80–82]. In line with earlier water stress studies [8, 39, 83] eight days following the beginning of water stress, accumulation of ABA and up-regulated ABA-related transcripts were observed. Coincidingly, a large number of transcription factors were induced by drought including ARFs. The role of ARF in abiotic stress response remains largely unexplored compared to development. However, cumulative evidence suggests that ARFs may be implicated in integrating auxin and ABA responses in environmental responses especially during drought stress [84]. In line with previous evidences, this study suggests that ARFs may be involved in the transcriptional responses to drought.

In general, the parallel transcriptional and metabolomic analysis showed a coordinated modulation of leaf metabolism under water deficit. Earlier comparative study of the effect of water deficit and salinity on grapevine leaf have shown the major stress effects in relation to ROS and energy metabolism, transcription factors, ABA and osmolite concentration [8]. The authors pointed out higher linkage in accumulation of stress related metabolites and gene expression involved in photosynthetic, gluconeogenic, and photorespiratory pathways and suggested the functional significance in adjusting cellular osmosis, ROS detoxification and photoinhibition. The genome-wide transcriptional study conducted by [26] also indicates global reprogramming of cellular metabolism as an adaptation to water deficit. In addition to an overall support to previous studies of grapevine response to stress, the present season-long study suggests that grapevine acclimation encompasses a multilevel coordination between the whole plant physiology, leaf area dynamics and temporally distinct modulation of leaf metabolism.

## Conclusions

The integrative leaf physiology, transcript and metabolite analysis under partially controlled field condition enabled us to examine stress temporal effect on grapevine leaf metabolism. Progressive water stress caused a sequential stress response from gene transcript-metabolite alteration, osmotic adjustment to leaf shedding and metabolic relaxation. The progressive leaf shedding and the consequential reduction in transpiration rates led to a rise in leaf water potential as the season progressed. The activation of stress associated responses, e.G. *aba* responsive transcription factors, at the early stage was followed by stress related responses such as increased osmolite concentration, (eg.,Ca^2+^ ion), upregulation of ROS metabolism and stress associated gene transcripts and corresponding metabolites such as GABA, Proline, myo-inositol, galactinol and raffinose.

## Methods

### Experiment design and plant growth conditions

The experiment was conducted on 4-years old Merlot vines grafted onto SO4 rootstock at the Udine University experimental farm (North of Italy, 46° 02’ N, 13° 13′ E; 88 m a.s.l.) during 2014 and 2015 growing seasons exactly as describe in [23, 34]. To mimic the open field condition while preventing natural precipitation, the vines were grown under 4.5 m high tunnel opened from all sides but covered with plastic film (EVA, ethylene-vinyl-acetate) on the top as described in [85]. Each vine was grown in 45 l container placed on a scale (experimental plant) in order to get daily lysimeter evapotranspiration values (ET_LYS_, [86]). The side of the pot was covered with aluminum foil to avoid excessive heat build-up. The pots were filled with a mixture of 49.0% sand, 31.5% silt, and 19.5% clay supplemented with 20% perlite. The vines were cane pruned to a single Guyot (0.8 m high), with 8 to 10 nodes per cane, and trained to vertical shoot positioning. The irrigation method, mineral nutrition, and fungicide treatments were applied as described in [23].

Two irrigation treatments were applied starting from veraison (50% color change)– well watered (120% of ET_LYS_, WW) and water deficit (35% of ET_LYS_ WW plant, WD) with four replicates applied in randomized manner along the row of pots as described in [23]. Three days after the treatment initiation, the soil water content (θ) was significantly different between the treatments, and at day 10 the minimum θ was reached and then kept similar for the remainder of the experiment (detailed description in [23]). Results of the first season are given in details when a consistent pattern of change exists in the two seasons. Seasonal differences are reported in the supplementary results.

### Leaf area measurement

The leaf area of experimental vines was evaluated five times during the experiment by a linear model relating the leaf length (measured for all leaves of four plants per treatment) and the leaf area as described in [23].

### Leaf physiology measurements

Leaf water potential (Ψ_l_; MPa) was periodically measured at midday during clear sunny days using a pressure bomb chamber (Soil Moisture Co., Santa Barbara, CA, USA) as previously described [10]. Pressure-Volume (P-V) measurements were conducted following the protocol of [87] on 6 adult leaves per treatment to understand the osmotic concentration. Gas exchange was measured in parallel to the water potential on sun exposed and fully expanded leaves using a LI-6400 Gas Exchange System (LICOR, Lincoln, NE) under constant light intensity (1000 μmol.m^− 2^ s^− 1^) and CO_2_ concentration (400 μmol.mol^− 1^) at ambient humidity and leaf temperature. The same leaf used for gas exchange measurements was then sampled for the extraction of metabolites and transcripts as described below.

### Leaf sampling and metabolite extraction

Samples for metabolic analysis were taken at nine time points in 2014 (− 1 (one day before deficit irrigation imposition), 1, 4, 8, 13, 18, 28, 35 and 53 days following deficit irrigation imposition) and six time points in 2015 (− 1, i.e.one day before deficit irrigation imposition 1, 3, 6, 12 and 26 days following deficit irrigation imposition). At each sampling time point, young, sun exposed and fully expanded leaf samples were taken, divided into two portions and used for metabolite and transcript analysis at the respective sampling stages. Each portion was immediately wrapped in aluminum foil and snap frozen with liquid nitrogen, and then kept at − 80 °C until further analysis. Sample preparation, tissue grinding and metabolite extraction for parallel GC-MS and LC-MS analysis were performed exactly as previously described [88]. Samples for GC–MS analysis were processed following the method of [89, 90]. All chemicals were purchased from Sigma Aldrich if not indicated otherwise. Frozen powder of 30 mg tissue was incubated in a 1 ml pre-chilled methanol/water/chloroform extraction mixture (2.5/1/1 *v*/v/v). The extraction mixture contains internal standards ribitol (i.e. 0.2 mg/ml in water), ampicillin (1 mg/ml in water) and corticosterone (1 mg/ml in methanol) to give a concentration of 0.86 mg ml^− 1^, 5.6 mg ml^− 1^, 7.16 mg ml^− 1^, respectively in the final injected volume. The sample-extraction mix was then briefly vortexed, centrifuged for 2 min at 14,000 RPM (microcentrifuge 5417R) and the supernatant was decanted into the new tubes. The supernatant was mixed with 300 ml of chloroform and 300 ml of UPLC-grade water, briefly vortexed and then centrifuged at 14,000 RPM for 2 min. For GC-MS analysis, 100 μl of extract from the water/methanol phase was dried in a vacuum concentrator (Eppendorf Concentrator Plus) for derivatization [89]. The remaining water/methanol phase was transferred to UPLC vials for LC-MS analysis.

### GC-MS derivatization and analysis

Samples for GC–MS analysis were derivatized exactly as described previously [88]. The sample set also included a quality control reference comprising *Arabidopsis thaliana* from a bulked extraction of *Arabidopsis thaliana* Columbia-0 plants and a mixture of authentic metabolite standards (0.05 mg/ml). A volume of 1 μl was then injected into the GC column in a splitless mode or with a split ratio of 50:1. Spectral searching, based on the National Institute of Standards and Technology (NIST, Gaithersburg, MA, USA) algorithm incorporated in the Xcalibur® data system (version 2.0.7), was done against retention index (RI) libraries downloadable from the Max-Planck Institute for Plant Physiology in Golm, Germany [91] and normalized by the internal standard ribitol. A spiking method was used to distinguish between metabolites with very similar RI and spectrum (e.g., rhamnose and fucose).

### LC-MS condition and data processing

For LC-MS analysis, 2 μl of extracted sample was injected onto a UPLC-QTOF-MS system equipped with an ESI interface (Waters Q-TOF XevoTM: Waters MS Technologies, Manchester, UK) operating in negative and positive ion modes. The chromatographic column conditions, solvent composition and gradient program were maintained exactly as described in [92]. All analyses were acquired using leucine enkephalin for lock mass calibration to ensure accuracy and reproducibility, at a concentration of 0.4 ng L^− 1^, in 50/50 of acetonitrile/H_2_O with 0.1% v/v formic acid. The MS conditions were set essentially as described in [69]. UPLC data processing MassLynxTM software (Waters) version 4.1 was used as the system controlling the UPLC and for data acquisition as described in [69]. The raw data acquired were processed using MarkerLynx application manager (Waters) essentially as described previously [69]. Metabolites were also identified based on standards, fragmentation pattern searched against the Chemspider metabolite database (http://www.chemspider.com/) and further confirmed with previous metabolite annotations [93–99].

### RNA extraction, RNA-seq, and cis-regulatory element analysis

Transcriptomics data was analyzed only for the early time points (i.e. 4 and 8 days after treatment; DAT) during the 2014 season, while one time point from the 2015 season (i.e. 12 DAT) was used as confirmatory data.

Frozen leaf was ground in liquid nitrogen and total RNA was extracted from 100 mg tissue using Sectrum™ plant total RNA kit (Sigma-Aldrich, STRN50). The quality and concentration of extracted RNA was determined using Bioanalyzer Chip RNA 7500 series II (Agilent, Santa Clara, CA) and a Nanodrop 2000 spectrophotometer (Thermo Scientific, Wilmington, DE). Following quality assessment, library construction was conducted using TruSeq RNA Sample Prep Kit v2.0 (Illumina). Sequencing was performed on an Illumina HiSeq 2000 sequencer (Illumina) at IGA Technology Services (Udine, Italy). Quality check, filtering, and trimming of raw sequence reads (50 nt, single-end) was performed using trimmomatic v0.36 [100] with the following parameters; leading, trailing, avgqual, and minlen parameter values are 20, 20, 20, and 40, respectively. Other parameters were default. The resultant reads were aligned to the reference *Vitis vinifera* genome (12x) [101] using bowtie2 default parameter (v2.2.7) [102]. Read summarization was performed with a HTSeq-count (v0.6.1) with default settings [103] using the 12xV1 *Vitis vinifera* annotation (GTF format) file. Differential expression (DE) analysis was conducted with DESeq2 [104]. A false discovery rate (FDR) < 0.05 and an absolute shrunken log2 fold change > 1 defines differentially expressed genes within each comparisons. Gene expression abundance was calculated using DESeq2 and represented as Reads Per Kilobase of transcript, per Million mapped reads (RPKM) and variance stabilized transformed (VST). Principal component of the transcriptome dataset (29,970 genes) was performed using the VST-transformed transcript abundance.

Gene ontology (GO) enrichment analysis of DE genes were performed with BinGO [105] using the GO associations available from the latest 12x V1 functional annotation [106]. Plant GO SLIM categories were considered. Categories are significantly enriched at FDR < 0.05 as determined by Fisher’s exact test adjusted with FDR for multiple testing corrections. Enrichment for cis-regulatory element (CRE) in promoters (1 kb region upstream of the 5’ UTR or transcriptional start site) of DE genes were determined using hypergeometric test adjusted with false discovery rate (FDR) correction with 222 CREs ranging between 6- to 8-mers according to the procedure of [107]. A stringent cut-off of FDR < 0.01 denotes significantly enriched CRE.

### Leaf inorganic ions concentration measurement

Inorganic ions were extracted from leaf samples at (8, 18 and 53 DAT) using 20 mg of ground sample with 15 ml distilled water, boiled at 100 °C for 5 min, kept at orbital shaker for about 1 min, and filtered through a cellulose nitrate filter [24]. The ion content was determined using an inductively coupled Plasma Emission Spectrometer. The contribution of the different inorganic ions and soluble sugars to osmotic adjustment were calculated as previously described in [24].

### Statistical analysis

For metabolite analysis, the normalized data set (to tissue dry weight and internal standards) was subjected to Student’s t-test to compare between treatments at each sampling data point. Principal component analysis (PCA) was carried out on log_10_ transformed data using TMEV software package [108]. Results of the first season are given in details when a consistent pattern of change in the two seasons was found. Seasonal differences are reported in a supplementary results description.

## Additional files

Additional file 1: **Table S1.** Stomatal conductance measurements of 2014 and 2015 presented as mean (*N* = 4) and standard error. The respective time points are indicated with days from treatment application. **Table S2.** Fold change (Treatment/Control) of leaf secondary metabolites during the time course of the experiment in 2014 and 2015. **Table S3.** Fold change (Treatment/Control) of leaf central metabolites during the time course of the experiment in 2014 and 2015. **Table S4.** Summary of RNA sequencing analysis metrics. The table show the number of filtered reads and aligned reads (filtered) to the 12X grapevine reference genome that are counted. **Table S5.** Transcript abundance of the all 29,970 genes, reported as Reads Per Kilobase of transcript, per Million mapped reads (RPKM), in leaves of well-watered and water deficit treated grapevines at day 4, 8 (2014) and day 12 (2015). **Tables S6–S8.** Summary of differentially expressed genes (FDR < 0.05, |log2 FC| > 1) in grapevine leaves comparing well-watered and water deficit treatments. DESeq2 statistical outputs of day 4, 8 (2014) and day 12 (2015) treatments are listed in Table S6, S7, and S8, respectively.. Annotation of genes were referred from Vitisnet (https://www.sdstate.edu/agronomy-horticulture-and-plant-science/functional-genomics-bud-endodormancy-induction-grapevines-5) (Grimplet et al. 2009). **Table S9.** List of differentially expressed genes in grapevine leaves that are common and unique leaves in each intersection of the Venn diagram described in Fig. 4b. **Table S10.** Summary of enriched (FDR < 0.05) plant GO slim categories in grapevine leaves comparing well-watered and water deficit treatments at day 4, 8 (2014) and day 12 (2015). Other statistical outputs of BiNGO (Maere et al. 2005) are reported. **Table S11.** Summary of cis-regulatory elements (CRE) enriched at a threshold of FDR < 0.01 in promoters of drought-regulated genes at day 4, 8 (2014) and day 12 (2015). For each treatment group, the number of genes containing the CRE, group size, total number of genes in the genome containing the CRE, promoter size, enrichment *P*-value, FDR, and lists of genes containing the CRE with their CRE position along the gene promoter are reported. (XLSX 3596 kb)

Additional file 2: **Figure S1.** Number of significantly altered metabolites under water stress identified using GC-MS (primary metabolites) and LC-MS (secondary metabolites) during the course of the experiment. (PPTX 38 kb)

## Abbreviations

3-PGDH: 3-phosphoglycerate dehydrogenase
4CL: 4-hydroxycinnamoyl CoA ligase
6PFK: 6-phosphofructokinase
A-Gal: alpha-galactosidase
Asp-AT: aspartate aminotransferase
AspK: aspartate kinase
AspK: aspartate kinase
AspO: aspartate oxidase
BPM: bisphosphoglycerate mutase
C4H: cinnamate 4-hydroxylase
CAD: cinnamyl alcohol dehydrogenase
CCR: cinnamoyl-CoA reductase
CHI: chalcone isomerase
CHS: chalcone synthase
CM: chorismate synthase
COMT: caffeic acid methyltransferase
DFR: dihydroflavonol-4-reductase
DHQS: dehydroquinate Synthase
F16BP: fructose-1,6-bisphosphate
F3H: flavonoid 3′ hydroxylase
F5H: ferulate 5-hydroxylase
FBA: fructose bisphosphate aldolase
FK: fructo kinase
FLS: flavonol synthase
G3PD: glyceraldehyde-3-phosphate dehydrogenase
G6PI: glucose-6-phosphate isomerase
GABAT: gamma amino butyric acid transaminase
GAD: glutamic acid decarboxylase
GlnS: glutamine synthase
GluDH: glutamate dehydrogenase
GluS: glutamate synthase
GolS: galactinol synthase
HCT: hydroxycinnamoyl CoA
HXK: hexokinase
INV: invertase
OAT: orinthine aminotransferase
P5CS: pyrroline-5-carboxylate reductase
PAL: phenylalanine ammonia lyase
PK: pyruvate kinase
PPH: phosphopyruvate hydratase
RafS: raffinose synthase
RP3E: ribulosephosphate 3-epimerase
Ser-Gly-AT: serine/glycine acetyltransferase
Ser-OAT: serine acetyltransferase
TrpS: tryptophan synthase
