# Supplementary figures and images for: Swift metabolite changes and leaf shedding are milestones in the acclimation process of grapevine under prolonged water stress
Source: BMC Plant Biol. 2019 Feb 11;19:69. doi: 10.1186/s12870-019-1652-y (PMC6371445; doi:10.1186/s12870-019-1652-y)

## Slide 1
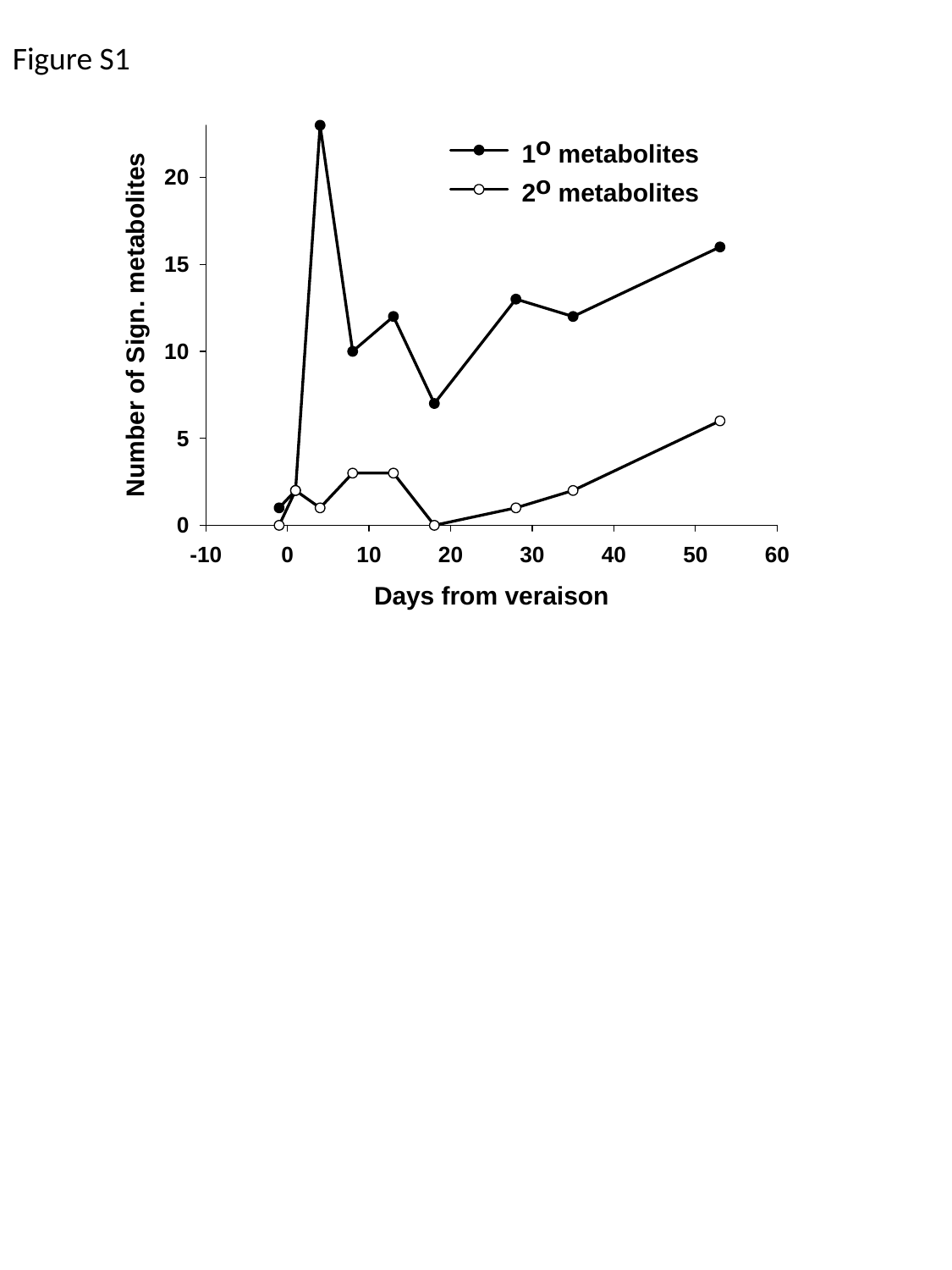

Figure S1

Supplement: Supplementary file 2 — Figure S1. Number of significantly altered metabolites under water stress identified using GC-MS (primary metabolites) and LC-MS (secondary metabolites) during the course of the experiment. (PPTX 38 kb) [file 12870_2019_1652_MOESM2_ESM.pptx]
